# Trusted Information Sources About the COVID-19 Vaccine Vary in Underserved Communities

**DOI:** 10.1007/s10900-023-01319-0

**Published:** 2024-02-01

**Authors:** Brian R. Benson, Syed A. Rahman, Jacob Bleasdale, Shunlei Win, Kaylyn Townsend-Kensinger, Matthew Cole, Kabir Jalal, Jihnhee Yu, Gene D. Morse, James L. Mohler, Rolanda L. Ward

**Affiliations:** 1 University at Buffalo, Buffalo, NY USA; 2 Department of Social Work, Niagara University, Niagara Falls, NY USA; 3 Frontier Science, Amherst, NY USA; 4 Roswell Park Comprehensive Cancer Center (Urology, Pharmacology and Therapeutics), Buffalo, NY USA

**Keywords:** Western New York, COVID-19 Vaccine, Vaccine Hesitancy, Underrepresented Populations

## Abstract

The COVID-19 pandemic was one of the deadliest global public health events. In the United States, over 1.1 million individuals have died, and now COVID-19 is the third leading cause of death (CDC, 2023). Vaccine uptake has stalled among different demographics. Vaccine hesitancy, a delay in accepting or refusing vaccines, poses a significant challenge regardless of the availability of safe and effective COVID-19 vaccines. This study aimed to identify disparate COVID-19 vaccine uptake among individuals in Western New York. The primary objective was to identify the factors contributing to lower rates of COVID-19 vaccination within this population.

Data were collected from 585 adults recruited from 20 Niagara and Erie Counties sites using a self-administered survey on vaccine hesitancy, vaccination status, and COVID-19-related characteristics. The survey included the adult Vaccine Hesitancy Scale (aVHS) and acquired information on demographic characteristics and COVID-19 impact, knowledge, and information sources. Data were analyzed using descriptive statistics, a chi-squared test, a Wilcoxon rank-sum test, and a logistic regression model.

Findings suggest that unvaccinated participants (n = 35) were concerned about vaccine side effects (48.6%). For vaccinated/unboosted participants (n = 52), they (40.0%) reported clinical concerns. After adjusting for gender and age, healthcare provider guidance and family guidance remained significant predictors of vaccination status, while clinical research studies were significant predictors of booster status. Findings from this study suggest public health interventions that target vaccine education and facilitate well-informed decisions about COVID-19 vaccines lead to less vaccine hesitancy.

**Supplementary Information:**

The online version contains supplementary material available at 10.1007/s10900-023-01319-0.

## Background

Underrepresented populations have suffered disproportionately during the COVID-19 pandemic. Among racial and ethnic groups, Black and Latino populations have experienced a disproportionate impact [1]. Hospitalization and death were higher as vaccination rates remained low among underserved communities during early 2021 [2]. Hospitalization and death rates were likely driven by disparities caused by economic differences, which increased the risk of COVID-19 exposure and limited access to healthcare and COVID-19 vaccine uptake [3]. At the time of this study, the New York State Vaccine Tracker (2023) reported that more than 70% of Western New York adults were vaccinated with at least one dose, but the rate is lower among Blacks (53.1%) [4, 5].

The Food and Drug Administration granted Emergency Use Authorization for the Moderna and Pfizer-BioNTech COVID-19 vaccines in response to the pandemic in late 2020 [6]. The population-level effectiveness of these vaccines relied on widespread uptake. Estimates suggested that more than 70% of the U.S. population would need to be vaccinated to achieve herd immunity [7]. However, vaccine hesitancy persisted among individuals despite numerous research studies demonstrating the safety and effectiveness of COVID-19 vaccines [8, 9]. The World Health Organization (WHO) defined vaccine hesitancy as “a delay in acceptance or refusal of vaccines despite their availability” [10]. Vaccine hesitancy encompasses a range of attitudes, beliefs, and behaviors that may vary across different vaccines, individuals, and communities [10]. The WHO identified vaccine hesitancy as one of the top ten major threats to global health [11]. Therefore, the reasons behind vaccine delay or refusal must be understood and addressed to increase vaccine uptake, particularly among groups disproportionately affected by the pandemic [12].

Understanding the factors contributing to COVID-19 vaccine hesitancy among underrepresented populations is essential for effective public health initiatives [13]. Vaccine hesitancy poses a significant challenge to widespread vaccine uptake and mitigating the impact of the pandemic [14]. This study investigated the primary motives and information sources associated with vaccine hesitancy in the Western New York region’s underserved communities of Erie and Niagara counties. Western New York consists of 8 counties with a high proportion of underserved residents living in rural neighborhoods and the cities of Buffalo and Niagara Falls. This study used a non-probability sampling method, convenience, to recruit underserved communities to understand concerns about COVID-19 vaccine safety and the role of government and healthcare providers as information sources. These insights could help address vaccination barriers, promote equitable vaccine uptake in underrepresented populations, and ultimately control the spread of COVID-19.

### Objectives

#### Primary Objective

The primary objective was to identify areas of disparate vaccine uptake and identify the factors that may have contributed to lower vaccination rates. The target was zip codes with disparate vaccination rates in Western New York, which may include people of color and urban and rural participants living in low-income communities.

#### Secondary Objective

Our secondary objective was to identify areas for further vaccine education within the broader community. The focus was to support the development of an informed and empowered community where people have access to trustworthy information, are aware of the advantages and safety of vaccinations, and are well-equipped to make informed decisions about their health.

## Methods

### Data Collection

Researchers identified zip codes where vaccination rates were low using data from the New York State Department of Health to inform the selection of locations for data collection. At each location, a convenience sample of adults was recruited to complete a survey regarding vaccine hesitancy. Data were collected from July 2022 - November 2022 at 10 churches, seven community centers, two community events, and a food pantry in Niagara and Erie Counties. Participants were eligible if they were at least 18 and could read, write, and speak English.

A research team member screened potential participants for eligibility using paper forms. If eligible, participants completed a self-administered survey and received a $25 Tops Friendly Market or Walmart gift card. All study materials were stored in a locked cabinet at Niagara University’s Rose Bente Lee Ostapenko Center for Race, Equity, and Mission. The Institutional Review Boards at Niagara University and the University at Buffalo provided a waiver of written informed consent (STUDY0006009).

### Vaccination Hesitancy Survey

The survey used to collect the vaccine data was created following a study revision. Revisions were made to the inclusion criteria and resulted in the removal of a qualitative focus group. Qualitative questions were added to the survey to replace the focus group. Modifications to the inclusion criteria allowed all adults 18 years of age or older to participate regardless of vaccination status. The survey was administered using paper forms and collected anonymously (Appendix 1).

Data were collected on various demographic characteristics, including age, race, ethnicity, and gender identity. Survey participants voluntarily shared their vaccination status and attitudes toward the COVID-19 vaccine. Our survey reproduced the Adult Vaccine Hesitancy Scale (aVHS) that was developed by the WHO’s Strategic Advisory Group of Experts (SAGE) [14]. aVHS consists of 10 items that assess various aspects of vaccine hesitancy on a 5-point Likert scale ranging from “least hesitant” to “most hesitant” [15].

### COVID-19 Related Questions

Data about the receipt of the COVID-19 vaccine were collected in response to yes/no questions. Various questions were included to understand the impact of COVID-19 on the health and well-being of participants and their families (e.g., severe illness, most medically impacted, death due to COVID-19), knowledge of COVID-19, and trusted sources for COVID-19 related information. If participants responded affirmatively to any question, they were asked to describe their answers using an open-response format.

### Data Management

Collected data were entered by research assistants into an Excel spreadsheet created by Frontier Science Foundation (Amherst, New York). A data entry plan was established to ensure data entry accuracy. 30% of surveys were randomly selected to ensure accuracy between paper surveys and the Excel spreadsheet. The research team resolved all discrepancies. Two survey questions demonstrated a significant trend of error, so all responses to these questions were reviewed to ensure accuracy. Cleaned data were imported into the Research Electronic Data Capture (REDCap) database. A total of 87 surveys did not include the demographic characteristics due to revisions made to the survey instrument during initial data collection, which resulted in 498 complete surveys and 87 incomplete surveys.

### Statistical Analysis

Descriptive statistics, which included mean, standard deviation, median, and interquartile ranges, were estimated for continuous measures, such as vaccine hesitancy score and age. In contrast, frequency counts and odds ratios were estimated for categorical responses, such as COVID-19 vaccine status, vaccine hesitancy status, gender, race, ethnicity, and individual item responses.

Wilcoxon rank-sum tests were calculated for continuous measures and chi-square and Fisher’s exact tests were calculated for categorical measures to test the association between COVID-19 vaccine status and vaccine hesitancy status [16–18]. A logistic regression model was used to model the probability of receiving the COVID-19 vaccine as a function of hesitancy, race, gender, age, population density, and income, and to model the probability of vaccine hesitancy as a function of race, gender, and age [19].

Individual survey responses were compared. Responses to survey question 22 (Select ALL the reason(s) for why you have decided not to receive the vaccine at this time.) and question 29 (Select ALL the reason(s) why you have decided not to receive a vaccine booster at this time.) were compared to evaluate differences between vaccinated/unboosted and unvaccinated individuals using chi-squared tests, odds-ratios, and relative risks, and vaccinated and unvaccinated respondents to questions 24 (Select ALL information sources that would help you decide to get vaccinated.) and 27 (Select ALL information sources that assisted you in deciding to get your first vaccine:) were compared using the same measures [19]. Responses to questions 24, 27, and 31 (Select ALL information sources that will help you make the decision to receive the vaccine booster.) were grouped according to information source (institutional, experiential, or both) and compared against vaccinated/unvaccinated and vaccinated but unboosted/unvaccinated groups using a chi-square test [20].

A logistic regression model was used to model item responses to questions 24/27 as a function of vaccine status, gender, and age, and to model responses to questions 22/29 as a function of unvaccinated/vaccinated but unboosted status, gender, and age [19].

## Results

A total of 599 surveys were collected (302 in Niagara County and 297 in Erie County) at 20 sites (11 in Niagara County and nine in Erie County). Table 1 (n = 483) provides descriptive statistics for the sample of participants who completed the surveys. A discrepancy exists between the total number of participants and the available demographic data. Demographic questions were not available for approximately the first 90 surveys but were available for the remaining participants. 2% (n = 14) of survey responses were excluded due to ineligibility responses on surveys that yielded a final sample size of 585. The research team later identified inaccuracies in participant reporting that resulted in the exclusion of many participants. Reasons for exclusion included vaccinated participants responding to questions meant for unvaccinated participants or vice versa. The resulting sample size for analysis was 359 vaccinated and 40 unvaccinated participants. Researchers note that in some cases, sample sizes smaller than 399 can be used by screening the responses for quality and accuracy.

**Table 1:** Demographic Characteristics of Survey Participants (n = 483)

	Total	Unvaccinated	Vaccinated
Gender			
Female	303	44	259
Male	172	31	141
Transgender	3	-	3
Non-conforming, non-binary, or genderqueer	1	-	1
Agender	1	-	1
I Prefer not to answer	1	-	1
Other	2	-	2
Age			
18–24	40	7	33
25–34	65	16	49
35–44	80	23	57
45–54	88	9	79
55–64	102	8	94
65–74	69	6	63
75+	27	3	24
Race			
American Indian or Alaskan Native	14	-	14
Asian	9	1	8
Black or African America	264	35	229
Native Hawaiian or other Pacific Islander	4	2	2
White	128	22	106
I Prefer not to answer	11	2	9
Multiple Races	31	11	20
Other	20	2	18
Ethnicity (Hispanic/Latino/Latina/Latinx)			
Yes	42	5	37
No	384	59	325
I prefer not to answer	21	6	15

When modified for vaccination status by race, 84% of participants were vaccinated. The highest vaccination rates were among “other” (90%), Asian (89%), Black/African American (87%), and White (83%). In the early phases of the pandemic, Black communities had a lower vaccination rate compared to Whites. This analysis counters this narrative. No significant differences were found in vaccination rates by race (*p* = 0.248), thus race was not used for model adjustment.

Motives behind individuals who were initially unvaccinated versus those who were initially vaccinated were examined but did not receive booster shots later, aiming to identify any differences in their reasons. The primary motive for abstaining from vaccination or boosting (Table 2) differed in one area (*p* = 0.0049). Among the 35 participants who had not received any vaccine, 48.57% cited the concern that vaccination “will cause me harm.” Among the 52 participants who were vaccinated but had not received a booster, only 19.23% were concerned with harm. No differences were found for any of the other 8 items.

**Table 2:** Reasons Participants Chose to be Unvaccinated (n = 35) or Vaccinated/Unboosted (n = 52)

	Q22: Unvaccinated (N = 35)		Q29: Vaccinated/Unboosted (N = 52)								
Survey Items	Yes	Percent	Yes	Percent	OR	95% LCL	95% UCL	RR	95% LCL	95% UCL	*p*-value
It will cause me harm	17	48.57%	10	19.23%	3.97	1.38	11.61	2.53	1.31	4.85	0.0049
It is too expensive	2	5.71%	5	9.62%	0.57	0.05	3.76	0.59	0.12	2.89	0.6968
COVID-19 is a hoax	5	14.29%	6	11.54%	1.28	0.28	5.52	1.24	0.41	3.74	0.7499
It is unnecessary	10	28.57%	13	0.25	1.20	0.40	3.49	1.14	0.57	2.31	0.8056
It is not available where I live	-	-	-	-	-	-	-	-	-	-	-
I had trouble making an appointment	-	-	-	-	-	-	-	-	-	-	-
My family told me not to get it	5	14.29%	6	11.54%	1.28	0.28	5.52	1.24	0.41	3.74	0.7499
My friend(s) told me not to get it	5	14.29%	6	11.54%	1.28	0.28	5.52	1.24	0.41	3.74	0.7499
My healthcare provider(s) told me not to get it	1	2.86%	6	11.54%	0.23	0.00	2.02	0.25	0.03	1.97	0.2341

**OR**: Odds Ratio; **RR**: Relative Risk; **Q22**: Select ALL the reason(s) why you decided not to receive the vaccine; **Q29**: Select ALL the reason(s) why you decided not to receive a vaccine booster

Out of the sources of information that participants used to decide whether to get vaccinated or not, four sources of information differed between 359 vaccinated and 40 unvaccinated participants (Table 3). In the analysis without covariate adjustment, the vaccinated participants preferred guidance from government sources (61.28% vs. 35.00%; *p* = 0.0020), healthcare providers (61.28% vs. 22.50%; *p* < 0.001), and family members (50.98% vs. 15.00%; *p* < 0.001), whereas the unvaccinated valued more “clinical experience” (40.00% vs. 16.16%; *p* < 0.001). Clinical experience refers to greater uptake of the vaccine among the general population. No differences were found for any of the five other items. After adjusting for gender and age (Table 4), vaccinated participants cited their decision to get vaccinated was led by guidance from their healthcare provider and family (*p* = 0.0011 and *p* = 0.0013, respectively). Unvaccinated participants wished to be provided with more clinical experience (*p* < 0.0001). Regarding the significance of the adjusted covariates, females preferred healthcare provider guidance (*p* = 0.0002) and younger individuals preferred personal stories (*p* = 0.002). No differences were found for the 5 other items.

**Table 3:** Helpful Information When Deciding to Get the COVID-19 vaccine by Unvaccinated (n = 40) and Vaccinated (n = 359) participants

	Q24: Unvaccinated (N = 40)		Q27: Vaccinated (N = 359)								
Survey Items	Yes	Percent	Yes	Percent	OR	95% LCL	95%UCL	RR	95% LCL	95% UCL	*P*-Value
Clinical research studies	19	47.50%	153	42.62%	1.22	0.60	2.47	1.11	0.79	1.58	0.6148
Government (such as FDA/CDC/Local health department) guidance	14	35.00%	220	61.28%	0.34	0.16	0.70	0.57	0.37	0.88	0.0020
More Clinical Experience	16	40.00%	58	16.16%	3.46	1.61	7.25	2.48	1.58	3.87	0.0008
Healthcare provider guidance	9	22.50%	220	61.28%	0.18	0.07	0.41	0.37	0.21	0.66	0.0000
Family guidance	6	15.00%	183	50.98%	0.17	0.06	0.42	0.29	0.14	0.62	0.0000
Friend guidance	9	22.50%	126	35.10%	0.54	0.22	1.20	0.64	0.35	1.16	0.1170
Information found on the Internet	12	30.00%	88	24.51%	1.32	0.58	2.82	1.22	0.74	2.03	0.4456
Celebrity endorsements	2	5.00%	32	8.91%	0.54	0.06	2.26	0.56	0.14	2.25	0.5572
Personal Stories	7	17.50%	104	28.97%	0.52	0.19	1.25	0.60	0.30	1.21	0.1400

**OR** = Odds Ratio, **RR** = Relative Risk; **Q24**: Select ALL information sources that would help you decide to get vaccinated; **Q27**: Select ALL information sources that assisted in you deciding to get your first vaccine

**Table 4:** Preferred Information Sources Adjusted for Gender and Age (n = 308)

	Received Vaccine		Female		Age	
Survey Items (24/27)	Estimate	*P*-Value	Estimate	*P*-Value	Estimate	*P*-Value
Clinical research studies	-0.4192	0.0594	-0.00786	0.2719	0.0853	0.4973
Government (such as FDA/CDC/Local health department) guidance	0.4061	0.064	-0.00591	0.4123	0.1254	0.3155
More Clinical Experience	-0.9593	< 0.0001	-0.0049	0.6153	-0.1116	0.5013
Healthcare provider guidance	0.8859	0.0011	0.0302	0.0002	0.00317	0.9812
Family guidance	0.9106	0.0013	-0.0119	0.1	0.1319	0.2942
Friend guidance	0.4199	0.1109	-0.00807	0.2783	-0.1749	0.1743
Information found on the Internet	-0.0806	0.7363	-0.00977	0.2333	-0.0999	0.4788
Celebrity endorsements	0.3564	0.4973	0.0231	0.0601	-0.0781	0.7101
Personal Stories	0.4588	0.1129	0.00409	0.6063	-0.4107	0.002

**Q24**: Select ALL information sources that would help you decide to get vaccinated; **Q27**: Select ALL information sources that assisted in you deciding to get your first vaccine

Information sources between unvaccinated and vaccinated/unboosted participants differed in one area after adjusting for gender and age (Table 5). Fewer vaccinated/unboosted participants chose clinical research studies as an important information source when deciding whether to get the vaccine (*p* = 0.0099). Regarding the significance of the adjusted covariates, females and younger participants preferred healthcare provider guidance (*p* = 0.017 and *p* = 0.0309, respectively).

**Table 5:** Preferred Information Sources to Help Get Vaccinated: Adjusted for Gender and Age (n = 62)

	Vaccinated/Un Boosted		Female		Age	
Survey Items (22/29)	Estimate	*p*-value	Estimate	*p*-value	Estimate	*p*-value
Clinical research studies	-0.7753	0.0099	-0.0119	0.5429	0.4744	0.1296
Government (such as FDA/CDC/Local health department) guidance	0.0183	0.9684	0.0241	0.3695	0.1333	0.7759
More Clinical Experience	-0.1739	0.6729	0.0234	0.3283	0.8673	0.1221
Healthcare provider guidance	0.2873	0.391	0.0541	0.017	-0.724	0.0309
Family guidance	0.3106	0.6112	0.0437	0.1389	5.6113	0.9421
Friend guidance	0.7517	0.0683	0.00512	0.8023	-0.128	0.6919
Information found on the Internet	0.0914	0.8396	-0.00343	0.8957	0.8036	0.1535
Celebrity endorsements	-0.4184	0.2432	0.0128	0.5724	0.3486	0.3676
Personal Stories	0.3556	0.5459	0.0369	0.1982	0.423	0.474

**Q22**: Select ALL the reason(s) why you decided not to receive the vaccine; **Q29**: Select ALL the reason(s) why you decided not to receive a vaccine booster

## Discussion

The cross-sectional surveys explored the factors contributing to COVID-19 vaccination hesitancy among medically underserved populations in Western New York. Participants were asked to indicate all factors influencing their decision to investigate the reasons behind the decision to forego vaccination or booster shots. A comparison between unvaccinated and vaccinated/unboosted participants indicated concern about the potential adverse effects of the COVID-19 vaccine. Of the participants who received the vaccination but were unboosted, 19.2% expressed concerns about potential harm. Similarly, almost half of the unvaccinated participants cited fear of harm as their reason for not receiving the vaccine.

The analysis explains the impact of information sources on participants’ decision-making regarding COVID-19 vaccination. Vaccinated individuals were more likely to rely on guidance from government agencies, healthcare providers, and family members. Over 60% of vaccinated participants considered information from government sources (such as FDA/CDC/local health departments) and healthcare providers as trustworthy and influential in their decision to get vaccinated. 51% mentioned family guidance as another influential factor. These findings underscore the importance of providing accurate and reliable vaccine information tailored to medically underserved populations.

Variations were observed in certain preferred information sources and reasons for vaccine acceptance among participants. Reasons reported by unvaccinated or vaccinated /unboosted participants included beliefs that “COVID-19 is a hoax” (14.3% [unvaccinated] vs. 11.5% [vaccinated/unboosted]), “my family told me not to get it” (14.3% vs. 11.5%), and “my friends told me not to get it” (14.3% vs. 11.5%). Among the vaccinated/unboosted participants, the reason not to receive the vaccine was “my healthcare provider told me not to get it” (11.5%), which was more than unvaccinated participants (2.9%). Information sources, such as guidance from friends, celebrities, and personal stories, were also noted, but no differences reached statistical significance. Females tended to rely more on the advice of healthcare providers. Older participants who reported being vaccinated preferred personal stories or experiences. These findings suggest that targeted communication strategies tailored to specific groups may address vaccine hesitancy.

This study offers important new information on the primary causes of COVID-19 vaccine reluctance in the Western New York region’s underserved areas. Participants who did not receive the vaccine showed concerns about potential harm, emphasizing the need for focused efforts to address safety concerns with accurate information. The differences in booster shot uptake among various groups emphasized the significance of targeted initiatives to encourage individuals to be up to date on all COVID-19 vaccines and boosters.

Addressing vaccine hesitancy within underserved communities requires a multifaceted approach that incorporates the direct influence of guidance from family and friends, personal experience, clinical research studies, government, and healthcare providers [8, 21]. Targeted interventions and communication strategies must be developed to increase vaccine uptake that focuses on the unique needs and concerns of underserved communities [22]. The main concern to address regarding COVID-19 vaccination is safety. Efforts to promote COVID-19 vaccination may be most effective when addressed by governmental agencies since 35.0% of unvaccinated and 61.3% of vaccinated participants chose this source of information as helpful when deciding to receive the vaccine or booster. Longitudinal studies are necessary to track the growth of hesitancy over time, especially for boosters where uptake has been low, while qualitative research can provide a deeper understanding of lived experiences that contribute to hesitancy [23]. This cross-sectional survey studied why someone may decide to receive the COVID-19 vaccine. However, it does not capture whether the individual decided to get the vaccine after completing the survey. During the time of data collection, Niagara and Erie counties implemented many services and programs, which included evidence-based informational materials and vaccination clinics. The researchers acknowledge that COVID-19 vaccination rates could have increased during this time. Public health campaigns should be tailored to address specific barriers and concerns faced by the Western New York population to improve communication and increase vaccine acceptance [22].

### Strengths and Limitations

The self-administered surveys allowed many participants to be involved at one time. Data collection required one or two individuals to distribute and collect the surveys. The survey questions were the same for each participant, which allowed researchers to collect the same data across a larger population of participants. The proportion of people with varying levels of vaccine hesitancy was estimated by surveying a diverse group of individuals in a relatively short period. Data were collected quickly (July 2022-November 2022), which seemed appropriate due to the urgency of the public health challenge posed by COVID-19. The survey allowed comparisons across different subgroups by potentially important factors that included age, gender, socioeconomic status, race/ethnicity, and zip code.

Data accuracy may be limited by the restriction in representation and participant recall. The sites chosen for data collection limited representation of all community members due to the selection of individuals who used or attended the services held when surveys were administered. The objective was to target zip codes with disparate vaccination rates in Western New York; however, the study revealed an urban-rural disproportion. Most participants lived in urban and suburban areas, limiting the generalizability to underserved rural communities. Participants’ ability to reflect on events or recall exact numbers may be limited by participant interpretation of questions. Responses provided by participants were believed to be due to question wording and repetitiveness. For example, answers varied from exceptionally high numbers to ranges of numbers when asked about illness or death caused by COVID-19. Therefore, longitudinal and qualitative studies are needed to understand the impact of interventions better and assess long-term vaccination outcomes [24, 25].

## Conclusions

Our cross-sectional survey provided insight into the causes of COVID-19 vaccination hesitancy in Western New York’s underserved population. Many participants who had not received vaccinations or booster doses cited concerns about potential harm as their rationale for not getting the immunizations. Information sources, such as advice from governmental organizations, medical professionals, and family members, played crucial roles in influencing the decisions of those who had received a vaccination. Vaccine hesitancy will be explored in more detail in our study population through a qualitative analysis, which will be published next. Our results should inform public health initiatives designed to remove obstacles and increase vaccine acceptability among the people of Western New York and may be applicable more widely.

### Electronic Supplementary Material

Below is the link to the electronic supplementary material.

Supplementary Material 1
