# Supplementary material for: Trusted Information Sources About the COVID-19 Vaccine Vary in Underserved Communities
Source: J Community Health. 2024 Feb 1;49(4):598–605. doi: 10.1007/s10900-023-01319-0 (PMC11306264; doi:10.1007/s10900-023-01319-0)
Supplement: Supplementary file 1 — Supplementary Material 1 [file 10900_2023_1319_MOESM1_ESM.pdf]

WNY COVID-19 Vaccine Hesitancy Survey

FOR OFFICE USE ONLY:

Record ID: ____________________________________

Site Location: ____________________________________

Meeting Date: ____________________________________

Participant Number: ____________________________________

INSTRUCTIONS: Please select the best answer.

1. Are you 18 years of age or older?

- ○ Yes
- ○ No

2. Can you read and speak English?

- ○ Yes
- ○ No

3. Have you received any vaccinations for COVID-19?

- ○ Yes
- ○ No

WNY COVID-19 Vaccine Hesitancy Scale

INSTRUCTIONS: Please rate your level of agreement with the following statements.

		Strongly disagree	Disagree	Neither agree nor disagree	Agree	Strongly agree
4a.	Vaccines are important for my health	○	○	○	○	○
4b.	Vaccines are effective	○	○	○	○	○
4c.	Being vaccinated is important for the health of others in my community	○	○	○	○	○
4d.	All routine vaccinations recommended by the CDC are beneficial	○	○	○	○	○
4e.	New vaccines carry more risks than older vaccines	○	○	○	○	○
4f.	The information I receive about vaccines from the CDC is reliable and trustworthy	○	○	○	○	○
4g.	Getting vaccines is a good way to protect me from disease	○	○	○	○	○
4h.	Generally, I do want my doctor or healthcare provider recommends about vaccines for me	○	○	○	○	○
4i.	I am concerned about serious adverse effects of vaccines	○	○	○	○	○
4j.	I do not need vaccines for diseases that are not common anymore	○	○	○	○	○

Additional Questions - **INSTRUCTIONS: Questions 5-8 ask about your knowledge of COVID-19. Please check all that apply and fill in the blanks.**

5. What do you know about SARS CoV-2, Coronavirus, COVID-19 pathogen? Please describe below:

____

____

6. Select ALL statements that represent what you know about COVID-19:

- ☐ COVID-19 is caused by a virus
- ☐ There is currently no cure for COVID-19
- ☐ Not everyone infected with COVID-19 will experience severe symptoms requiring hospitalization
- ☐ People with certain chronic diseases, such as cancer, diabetes, and heart disease, are more likely to suffer serious illness from COVID-19
- ☐ Vaccines against COVID-19 are safe and effective
- ☐ COVID-19 is spread through respiratory droplet/particles
- ☐ The risk of being infected with COVID-19 is reduced by wearing face masks
- ☐ The risk of being infected with COVID-19 is reduced by maintaining social distancing
- ☐ The risk of being infected with COVID-19 is reduced by frequently washing your hands
- ☐ Common symptoms of COVID-19 include one or more of the following: fever, cough, fatigue, trouble breathing, loss of taste/smell
- ☐ Testing for COVID-19 is available
- ☐ Other, see narrative above

7. Where did you learn your knowledge about COVID-19? Please describe.

____

____

8. Select ALL sources that provide you information about COVID-19:

- ☐ Government agencies, such as the CDC, FDA, and the local health department
- ☐ TV/Radio news
- ☐ Social media, such as Facebook, Twitter, and Instagram
- ☐ Internet (non-government websites)
- ☐ Family
- ☐ Friend(s)
- ☐ Healthcare provider(s)
- ☐ Other, see narrative above

INSTRUCTIONS: Questions 9-20 ask about your personal experience with COVID-19. Please select the best answer, check all that apply, and fill in the blanks.

9. Do you know anyone who has been medically impacted by COVID-19?

◯ Yes

◯ No

10. Please describe how people you know were medically impacted by COVID-19, if applicable:

____

____

11. Select ALL who were medically impacted by COVID-19:

- ☐ Spouse/partner/significant other
- ☐ Parent(s)
- ☐ Grandparent(s)
- ☐ Child(ren)
- ☐ Other family member(s)
- ☐ Friend(s)
- ☐ Co-worker(s)
- ☐ Neighbor(s)
- ☐ Healthcare provider(s)
- ☐ Other, see narrative above
- ☐ Not applicable

12. Has COVID-19 had a negative impact on the health of your family?

◯ Yes

◯ No

13. Please describe how your family's health was impacted, if applicable:

____

____

14. Select ALL who were impacted by COVID-19:

- ☐ I myself was sick with COVID-19
- ☐ My spouse/partner/significant other was sick with COVID-19
- ☐ My child(ren) was/were sick with COVID-19
- ☐ Other family member(s) was/were sick with COVID-19
- ☐ Incurred high medical bills
- ☐ Had to buy expensive medication/medical equipment
- ☐ Other, see narrative above
- ☐ Not applicable

15. Have there been serious illnesses or deaths in your family?

○ Yes

○ No

16. Please describe what happened if there was a serious illness or death in your family, if applicable:

____

____

17. Select ALL the individual(s) who were sick with COVID-19, if applicable:

- ☐ Spouse/partner/significant other
- ☐ Parent(s)
- ☐ Grandparent(s)
- ☐ Child(ren)
- ☐ Aunt(s)/Uncle(s)
- ☐ Niece(s)/Nephew(s)
- ☐ Cousin(s)
- ☐ Pet(s)
- ☐ Other, see narrative above
- ☐ Not applicable

18. Enter the number of individuals who were sick with COVID-19, if applicable:

____

19. Select ALL the individual(s) who died as a result of COVID-19, if applicable:

- ☐ Spouse/partner/significant other
- ☐ Parent(s)
- ☐ Grandparent(s)
- ☐ Child(ren)
- ☐ Aunt(s)/Uncle(s)
- ☐ Niece(s)/Nephew(s)
- ☐ Cousin(s)
- ☐ Pet(s)
- ☐ Other, see narrative above
- ☐ Not applicable

20. Enter the number of individuals who died:

____

INSTRUCTIONS: Questions 21-27 ask about receiving the initial COVID-19 vaccine. Please select the best answer, check all that apply, and fill in the blanks.

21. If you have not been vaccinated, why have you decided not to receive the vaccine at this time? Please describe.

____

____

22. Select ALL the reason(s) for why you have decided not to receive the vaccine at this time:

- ☐ It will cause me harm
- ☐ It is too expensive
- ☐ COVID-19 is a hoax
- ☐ It is unnecessary
- ☐ It is not available near where I live
- ☐ I had trouble making an appointment
- ☐ My family told me not to get it
- ☐ My friend(s) told me not to get it
- ☐ My healthcare provider(s) told me not to get it
- ☐ Other, see narrative above
- ☐ Not applicable

23. What kind of information will help you decide to get vaccinated? Please describe.

____

____

24. Select ALL information sources that would help you decide to get vaccinated:

- ☐ Clinical research studies
- ☐ Government (such as FDA/CDC/local health department) guidance
- ☐ More clinical experience
- ☐ Healthcare provider guidance
- ☐ Family guidance
- ☐ Friend guidance
- ☐ Information found on the Internet
- ☐ Celebrity endorsements
- ☐ Personal stories
- ☐ Other, see narrative above
- ☐ Not applicable

25. If you have received your first vaccination, why did you decide to receive the vaccine? Please describe.

____

____

26. What kind of information assisted your decision to get the first vaccine? Please describe.

____

____

27. Select ALL information sources that assisted you in deciding to get your first vaccine:

- ☐ Clinical research studies
- ☐ Government (such as FDA/CDC/local health department) guidance
- ☐ More clinical experience
- ☐ Healthcare provider guidance
- ☐ Family guidance
- ☐ Friend guidance
- ☐ Information found on the Internet
- ☐ Celebrity endorsements
- ☐ Personal stories
- ☐ Other, see narrative above
- ☐ Not applicable

INSTRUCTIONS: Questions 28-36 ask about receiving a COVID-19 booster vaccine. Please select the best answer, check all that apply, and fill in the blank.

28. If you have not received the vaccine booster, why have you decided not to at this time? Please describe.

____

____

29. Select ALL reason(s) why you have decided not to receive a vaccine booster at this time:

- ☐ It will cause me harm
- ☐ It is too expensive
- ☐ COVID-19 is a hoax
- ☐ It is unnecessary
- ☐ It is not available near where I live
- ☐ I had trouble making an appointment
- ☐ My family told me not to get it
- ☐ My friend(s) told me not to get it
- ☐ My healthcare provider(s) told me not to get it
- ☐ Other, see narrative above
- ☐ Not applicable

30. What kind of information will help you to make the decision to get the booster? Please describe.

____

____

31. Select ALL information sources that will help you make the decision to receive the vaccine booster:

- ☐ Clinical research studies
- ☐ Government (such as FDA/CDC/local health department) guidance
- ☐ More clinical experience
- ☐ Healthcare provider guidance
- ☐ Family guidance
- ☐ Friend guidance
- ☐ Information found on the Internet
- ☐ Celebrity endorsements
- ☐ Personal stories
- ☐ Other, see narrative above
- ☐ Not applicable

32. If you have received the vaccine booster, why did you decide to receive it? Please describe.

____

____

33. What kind of information assisted your decision to get a booster shot? Please describe.

____

____

34. Select ALL information sources that helped you make the decision to receive the vaccine booster:

- ☐ Clinical research studies
- ☐ Government (such as FDA/CDC/local health department) guidance
- ☐ More clinical experience
- ☐ Healthcare provider guidance
- ☐ Family guidance
- ☐ Friend guidance
- ☐ Information found on the Internet
- ☐ Celebrity endorsements
- ☐ Personal stories
- ☐ Other, see narrative above
- ☐ Not applicable

35. Who are the trusted sources that you go to for information? Please describe.

____

____

36. Select ALL sources you seek for information:

- ☐ Family
- ☐ Friends
- ☐ Healthcare provider
- ☐ TV/radio news
- ☐ Internet
- ☐ Government agencies, such as CDC, FDA, and the local health department
- ☐ Celebrities
- ☐ Social media
- ☐ Other, see narrative above
- ☐ Not applicable

INSTRUCTIONS: Questions 37 & 38 ask about your COVID-19 questions. Please check all that apply, or fill in the blank.

37. Are there any questions you would like to ask? Please describe below.

____

____

38. Select ALL questions you have about COVID-19:

- ☐ When will the COVID-19 pandemic end?
- ☐ When will life get back to normal?
- ☐ When will there be a cure for COVID-19?
- ☐ Why won't people take the COVID-19 vaccine?
- ☐ Why do people take the COVID-19 vaccine so readily?
- ☐ When will the COVID-19 vaccine be available near where I live?
- ☐ Will I have to get a COVID-19 vaccine booster shot?
- ☐ Will I have to get a COVID-19 vaccine booster shot more than once?
- ☐ Do I still have to wear a face mask?
- ☐ When can I stop wearing a face mask?
- ☐ Is it safe to visit friends/family?
- ☐ Is it safe to go to gatherings of more than 10 people?
- ☐ Other, see narrative above
- ☐ Not applicable

Demographics

INSTRUCTIONS: This section is all about you. Please fill in the blanks and choose the answer that best describes yourself.

1. **What is your age? _______**

2. **How do you identify your gender?**
   A. Female
   B. Male
   C. Transgender
   D. Non-conforming, nonbinary, or genderqueer
   E. Agender
   F. I prefer not to answer
   G. Other (please specify): _______________________________

3. **How do you identify your race?**
   A. American Indian or Alaskan Native
   B. Asian
   C. Black or African American
   D. Native Hawaiian or other Pacific islander
   E. White
   F. I prefer not to answer
   G. Multiple Races (please specify): _______________________________
   H. Other (please specify): _______________________________

4. **Do you identify as Hispanic or Latino/Latina/Latinx?**
   A. Yes
   B. No
   C. I prefer not to answer

5. **What Zip Code do you live in? _____________**

Thank you for completing the survey.
